# The impact of funding models on the integration of Ontario midwives: a qualitative study

**DOI:** 10.1186/s12913-023-10104-7

**Published:** 2023-10-11

**Authors:** Elizabeth K. Darling, Sylvie B. Lemay, ‘Remi Ejiwunmi, Katherine J. Miller, Ann E. Sprague, Rohan D’Souza

**Affiliations:** 1 McMaster Midwifery Research Centre, McMaster University, 1280 Main St. West, HSC-4H26, Hamilton, ON L8S 4K1 Canada; 2 Department of Obstetrics and Gynecology, McMaster University, Hamilton, ON Canada; 3 Department of Family Medicine, McMaster University, Hamilton, ON Canada; 4 Better Outcomes Registry and Network (BORN) Ontario, Children’s Hospital of Eastern Ontario Research Institute, Ottawa, ON Canada

**Keywords:** Midwifery, Integration, Remuneration, Reimbursement mechanisms, Fees, medical, Health services, Maternal health services, Ontario, Qualitative research

## Abstract

**Background:**

Nearly 30 years post legalisation and introduction, midwifery is still not optimally integrated within the health system of Canada’s largest province, Ontario. Funding models have been identified as one of the main barriers.

**Methods:**

Using a constructivist perspective, we conducted a qualitative descriptive study to examine how antepartum, intrapartum, and postpartum funding arrangements in Ontario impact midwifery integration. We conceptualized optimal ‘integration’ as circumstances in which midwives’ knowledge, skills, and model of care are broadly respected and fully utilized, interprofessional collaboration and referral support the best possible care for patients, and midwives feel a sense of belonging within hospitals and the greater health system. We collected data through semi-structured telephone interviews with midwives, obstetricians, family physicians, and nurses. The data was examined using thematic analysis.

**Results:**

We interviewed 20 participants, including 5 obstetricians, 5 family physicians, 5 midwives, 4 nurses, and 1 policy expert. We found that while course-of-care-based midwifery funding is perceived to support high levels of midwifery client satisfaction and excellent clinical outcomes, it lacks flexibility. This limits opportunities for interprofessional collaboration and for midwives to use their knowledge and skills to respond to health system gaps. The physician fee-for-service funding model creates competition for births, has unintended consequences that limit midwives’ scope and access to hospital privileges, and fails to appropriately compensate physician consultants, particularly as midwifery volumes grow. Siloing of midwifery funding from hospital funding further restricts innovative contributions from midwives to respond to community healthcare needs.

**Conclusions:**

Significant policy changes, such as adequate remuneration for consultants, possibly including salary-based physician funding; flexibility to compensate midwives for care beyond the existing course of care model; and a clearly articulated health human resource plan for sexual and reproductive care are needed to improve midwifery integration.

**Supplementary Information:**

The online version contains supplementary material available at 10.1186/s12913-023-10104-7.

## Background

### Introduction

The World Health Organization recognizes that there is a strong evidence base demonstrating that midwifery is essential to reducing maternal and newborn mortality and improving the quality of care in all settings, globally [1]. Almost 30 years after the legalisation and funding of midwifery and the establishment of university education programmes for midwives in Ontario, Canada, important challenges to the integration of midwives into the healthcare system still exist. Midwives cared for approximately 20% of pregnant people in Ontario in 2020–21 and this proportion continues to grow [2]. However, while midwifery services are available in more than 250 communities and at over 90 of 96 hospitals providing intrapartum services [2], restrictions on midwives’ scope of practice and access to hospital privileges^1^ for new midwives limit the contributions and overall growth of the profession in Ontario [3, 4]. Optimal integration of midwives is also hampered by a persistent lack of understanding about the profession and friction between provider groups which impedes interprofessional collaboration.

Several studies on midwifery in Ontario have identified that funding arrangements contribute to the challenge of midwifery integration into the health system and impede interprofessional collaboration [5–9]. In 2006, the Ontario Maternity Care Expert Panel (OMCEP) identified funding, along with aspects of legislation, regulation, and liability, as the elements that constitute “entrenched barriers to the ongoing delivery of high-quality maternity care” that were “limiting the implementation of positive change” and recommended changes including the creation of complementary funding schemes [7]. These identified barriers are congruent with our experience as midwifery, nursing, and physician obstetrical care providers. Although many recommendations from OMCEP have been realized, those around funding have not resulted in substantive change.

Our research addresses a gap in the literature regarding how funding policy and mechanisms for both physician and midwifery payment impact the integration of midwifery in the context of a universal healthcare system and a continuity of care midwifery model. Our overall objective was to understand the impact of funding arrangements on the provision of primary antepartum, intrapartum, and postpartum care services, including impacts on interprofessional collaboration (e.g., between family physicians, midwives, nurses, and obstetricians), the unintended consequences of funding arrangements, and the impact on the health system’s “quadruple aim” (improved population health, patient/client experience, provider experience, while keeping per capita costs manageable) [10]. The research question we address in this paper is how do funding arrangements impact the integration of the midwifery profession into Ontario’s health system.

### Context

The context for our research was the health system in Ontario, Canada’s largest province. The system includes publicly funded hospital, physician, and midwifery services. Citizens of Ontario or otherwise eligible residents are covered by Ontario’s Health Insurance Plan (OHIP). OHIP covers many emergency and preventative medical services and is funded through provincially-collected tax dollars [11].

Publicly funded hospitals in Ontario are not-for-profit organizations regulated through the Public Hospitals Act. This act lays out the governance structure for public hospitals, which involves a board of directors that has overall authority for the organization. Each board has a medical advisory committee (MAC), which is composed of elected and appointed members of the professional staff who are primarily or exclusively physicians [12]. Of note, nursing and midwifery representatives may be included and when present are non-voting members [12, 13]. Physicians play important roles such as chief medical officers and department chairs which yields them significant power in institutional decision-making. The MAC makes recommendations to the board regarding the appointment of medical staff, which include physicians, dentists, and midwives, all of whom must be granted ‘privileges’ in order to work within hospitals as independent practitioners (i.e., they are not employees of hospitals). Privileges allow these professionals to admit and discharge people to the hospital and to provide care to them while in hospital.

Care during pregnancy, labour and birth, and postpartum in Ontario is provided by family physicians, midwives, nurses, nurse practitioners, obstetricians, pediatricians, and anesthesiologists, with each of these provider groups playing varying roles. Family physicians, midwives, nurse practitioners, and obstetricians may all be the most responsible provider during the antepartum and postpartum periods, while only family physicians, midwives, and obstetricians act as the most responsible provider during the intrapartum period. Midwives and family physicians are generally low-risk intrapartum care providers. Obstetricians have expertise in high-risk care and act as consultants to midwives and family physicians, but also provide care for a large portion of low-risk births in Ontario. In rural settings with limited or no obstetrician services, family physicians may assume a more expanded scope and act as consultants to midwives. Family physicians, midwives, nurse practitioners, and pediatricians all provide newborn care. Nurses may be involved in the provision of antepartum or postpartum care in collaboration with physicians, and typically provide the majority of care in hospital during labour and the early postpartum period for people under the care of a physician, and in certain situations provide care to midwifery clients in hospital (e.g., following transfer of care to a physician). Anesthesiologists provide care during the intrapartum period.

Most people who give birth in Ontario receive initial antepartum care from their family physician or nurse practitioner (with approximately 50% of family doctors offering some antepartum or postpartum care) [14] and are then referred to an obstetrician for the remainder of their antepartum care in the second half of pregnancy. Access to care from an obstetrician in the first half of pregnancy is traditionally only available to people with high-risk pregnancies, with access varying by setting based on the availability of obstetricians. Only 8% of family physicians in the province provide intrapartum care, [14] and about 7.4% of people who give birth in Ontario receive all their antepartum and intrapartum care from a family physician [15]. Antepartum and postpartum physician care is primarily office-based and intrapartum care is all provided in hospital. Newborn care following a physician-attended birth is most often provided by a pediatrician or family physician, with use of pediatric services driven by parent choice and the availability of pediatricians. Additional services that may be available in some communities include hospital or public health run breastfeeding support and well-baby assessments programmes.

Roughly one in five pregnant people in Ontario receive care from a midwife [2]. Most midwives in Ontario work in group practices in midwifery-led continuity of care models which involve providing antepartum, intrapartum, and six weeks of postpartum care. In addition to office-based care, midwives provide early postpartum care to clients and their newborns in their homes. Intrapartum care is provided by midwives primarily in hospitals, but also at home or in birthing centres where such institutions exist. In 2021, 17.5% of midwifery clients gave birth outside of hospital [16]. In hospitals, midwives usually provide all aspects of low-risk intrapartum care (e.g., including care typically provided by nurses for physician patients). Nursing support occurs in specific circumstances (e.g., emergencies, precipitous births, and by arrangement in small and rural communities) and care is transferred to the physician-on-call and nursing staff when high risk or emergency situations out of the midwifery scope occur. Additionally, in some settings midwives are required by hospital policy or physician preference to transfer care for situations within their scope of practice (e.g., induction of labour or epidural pain relief).

### Funding arrangements

Midwifery care is funded through the Ontario Ministry of Health’s (MOH) Midwifery Program (OMP), which constitutes monies outside the OHIP funding pool. The great majority of midwives work in group practices and are remunerated for a bundle of services called a course of care (CoC), which includes all the care associated with one pregnancy and birth, from the first trimester to the end of six weeks postpartum, including newborn care. Midwives must provide at least 12 weeks of care or attendance at the birth to bill for a course of care. Midwifery funding is dispersed through regional transfer payment agencies that contract midwifery practices for a predetermined number of births each year [17]. In this remuneration structure, midwives are independent practitioners working as partners or associates within a midwifery practice group. Recently some alternative funding has been implemented in which midwives are salaried, which enables them to provide episodic care to meet community needs where the CoC model would not be feasible but where midwifery care would be beneficial [18]. This may take place within interprofessional teams or targeting high risk populations [18]. However, these projects are approved on a case-by-case basis and currently form only a small part of the work done by midwives in Ontario.

In contrast, family physicians and obstetricians are primarily funded to provide antepartum, intrapartum, and postpartum care through a fee-for-service (FFS) model.^2^ The majority of physicians are essentially small business owners who provide health services. FFS is payment for visits and for specific procedures done as defined by the OHIP Schedule of Benefits (SoB) [19]. This complex payment schedule is negotiated with the MOH by the Ontario Medical Association. There are no specific limitations for number of patients in care but there are limitations on the numbers of consultations and repeat procedures provided per patient (e.g., a physician can only bill for one consultation from a midwife per day for an individual patient). A day of providing antepartum care is much less lucrative than work on a birthing unit where a busy shift may equate to a very significant portion of a physician’s obstetric care related monthly income. Attendance at birth during evenings, nights and weekends is remunerated at 50–75% more than attendance during the day [19]. In many cases, the MOH gives hospitals monies to distribute stipends to certain specialists for being on-call^3^[20], however this stipend is only a small portion of remuneration and may be distributed at the department’s discretion [21, 22].

Hospital funding in the past has been decided at a regional level through Local Health Integration Networks (LHIN) [23]. However, the Ontario healthcare system is currently in transition with “Ontario Health” (OH) as the new lead agency with six approved geographical regions and Ontario health teams guiding delivery of health services [24]. Inpatient antepartum, intrapartum, and postpartum care funding comes from a hospital’s global budget which is based on a retroactive analysis of case mix in previous years. Hospitals have a level-of-care designation of 1, 2 or 3 depending on the number of births and the availability of intermediate and high-risk intrapartum and neonatal services [25], which are funded at different levels. Since funding is based on a retroactive analysis, hospitals are financially penalized if their program grows so that they provide more care than is funded in their budget; the funding mechanism does not consider the actual present need for intrapartum and neonatal services in the community a hospital serves or access to such care. Hospital budgets include monies for nursing and other allied health providers but not full compensation for the maintenance and renewal of equipment and buildings [26].

## Methods

### Theoretical framework

We approached the data using a constructivist approach to understand different perspectives (i.e.., there is no single truth; rather truth is relative and co-constructed by the researchers, individual, and society) [27, 28]. Our conceptual framework for data collection and analysis was shaped by two key concepts: the Quadruple Aim and Integration. The Quadruple Aim is a set of four factors that may be seen as the key objectives of a high performing health system, namely enhancing patient experience, improving population health, reducing costs, and improving the work life of healthcare providers [29–31]. We used the Quadruple Aim factors to inform our semi-structured interview guide. Within the context of healthcare, the term ‘integrated’ is commonly used to describe to care that supports collaboration between health professions to provide complete treatment to patients and improve overall wellbeing [32]. As Goodwin notes, “[m]uch depends on the ‘softer issues’ of relationship building and the ability to foster an environment where new collaborations and ways of working become accepted as the norm over time.” [33] We conceptualized optimal ‘integration’ of midwives as a set of circumstances in which midwives’ knowledge, skills, and model of care^4^ are broadly respected and fully utilized, interprofessional collaboration and referral are facilitated to support the best possible care for patients, and midwives feel a sense of belonging within hospitals and the greater health system. This conceptualization of integration informed our analysis of the data.

### Study design

We conducted a qualitative descriptive study using semi-structured interviews [34, 35]. This approach allowed us to synthesize the perspectives of a range of key stakeholders whose insights into the funding mechanisms for midwifery services specifically, and antepartum, intrapartum, and postpartum care more broadly, were based on direct experience. To triangulate these findings, we also conducted a documentary analysis of Canadian healthcare related websites. We obtained ethics approval (#8065) from the Hamilton Integrated Research Ethics Board in Hamilton, Ontario prior to commencing the research.

### Setting and participants

Our research focussed on the provincial funding arrangements for antepartum, intrapartum, and postpartum services within Ontario, Canada, including the funding of healthcare providers and facilities (e.g., hospitals). Participants in our study included four groups of healthcare providers (family physicians, midwives, nurses, obstetricians) and policy experts. Additional inclusion criteria were age over 18 years, ability to speak English, and familiarity with antepartum, intrapartum, and postpartum services in Ontario.

### Data collection

We used a multi-stage sampling approach to identify and recruit key informants from a range of settings across Ontario. We began with purposive sampling, in which members of the research team identified potential participants with the intention of ensuring representation from across the province from each profession, and variation in work experience with respect to sizes of communities and hospitals, including both rural and urban settings. We later used respondent driven sampling by asking participants to recommend others who they felt would offer valuable insight. We sent invitations to participate by email and followed-up with phone calls and/or emails one week after the initial invitation. After written informed consent was obtained, semi-structured one-on-one interviews were conducted by telephone using a semi-structured interview guide.^5^ Participants were asked about the impact of funding models on their work, interprofessional relationships with midwifery, unintended consequences of funding models, and the impact of funding arrangements on the Quadruple Aim (see Additional file 1: Interview Guide). All interviews were audio recorded and professionally transcribed.

For the documentary analysis, we searched the websites of Canadian and Ontario healthcare, medical, midwifery, and hospital organizations, and the Ontario Ministry of Health using midwifery and maternity care search terms followed by the addition of funding and/or integration to identify any web-page content and documents that pertained specifically to funding, financing, or remuneration.

### Data analysis

We conducted our analysis of the interview transcripts in Microsoft Excel, using an approach informed by our theoretical frameworks. Initially, we deductively organized data from the transcripts into categories related to impact of funding models based on our research objectives. We then used open coding to summarize and describe the data, followed by focussed coding to identify and categorize themes [34–36]. As we categorized the themes, we also examined the data to identify patterns, explanations, and interactions between emerging concepts and the participants, their profession, and their setting. Three researchers read and reread the transcripts and coded independently and then reviewed their results to ensure accurate data interpretation. Discrepancies were resolved through discussion among the research team. In keeping with common practice in qualitative inquiry, we began our analysis prior to completion of the data collection and continued interviews until there was evidence of data saturation [37]. Following analysis of the interview transcripts, one researcher reviewed and coded the documentary evidence, noting similarities and differences with respect to the interview findings.

### Reflexivity and Rigor

Our research team included representation from family medicine (KM), obstetrics (RD), nursing (AS), and midwifery (ED, RE, SL), and our interests in our research topic arose from our own work experiences and desires to support optimal healthcare for pregnant and birthing people and their families. To support participants’ comfort to openly express their thoughts, all but three interviews were conducted by a female PhD-trained health researcher with qualitative research experience who is a non-clinician and who had no prior relationship with the participants. To support triangulation of data collection, the remaining interviews were conducted by a female PhD-trained midwife researcher (ED) with qualitative research experience who was known to the participants but was not a professional colleague. Coding of the interviews was conducted by two masters-trained research coordinators, one who was a non-clinician and one who is a midwife (SL), and a PhD-trained midwife researcher (ED). The data analysis and coding were reviewed in detail by team members with the perspectives of medicine (RD) and nursing (AS) to ensure that the identified themes accurately reflected the data. Throughout the research process, we used self-reflection to maintain awareness of the influence of our professional perspective and openness to differing perspectives.

Rigor of our findings was supported through triangulation of data sources (different types of informants), methods (interviews and document analysis), and investigators. We also attended to the following criteria for rigor in qualitative research: credibility, authenticity, criticality and integrity [38], which are commonly applied to qualitative description [39]. To ensure credibility and authenticity, we worked to remain true to our research purpose and to the perspectives of our participants by collecting rich data and accurately representing it during analysis and reporting. By paying attention to our own influence during data collection and analysis, we remained true to the multiple perspectives shared by our participants. Using a non-clinician interviewer and an interprofessional research team helped to ensure that the voices of participants were not dominated by one particular perspective. Our iterative methods ensured criticality and integrity by incorporating recursive, repeated checks of our evolving interpretation and intentional searches for conflicting interpretations and opinions.

## Results

We invited 33 participants to participate in the study, of whom 20 completed interviews. Participants included midwives (*n* = 5), family physicians (*n* = 5), obstetricians (*n* = 5), nurses (*n* = 4), and a policy expert (*n* = 1). Three participants, all of whom were obstetricians, were male, and the rest were female or non-binary. Participants worked in a range of hospital settings where midwives work, including small level 1 hospitals, large level 2 community hospitals, and tertiary hospitals, across urban, rural, and remote contexts. Interviews were completed in January and February of 2020. Interviews took between 12 and 65 min (average length of 30 min).

Table 1 presents a summary of the key findings by healthcare provider group. Below, we describe our interview findings on the impact of funding arrangements on midwifery integration organized by the three main funding mechanisms we examined (midwifery, physician, and hospital), other notable findings, and proposed solutions. We then summarize the findings of our documentary analysis and briefly address how those findings aligned with our interview data.

**Table 1:** Key findings regarding Ontario funding arrangements for antepartum, intrapartum, and postpartum care by healthcare provider

Issue	Key Findings			
	**Family Physicians (FP)**	**Nurses (RN)**	**Obstetricians (OB)**	**Midwives (MW)**
Impacts of own funding arrangements on ability to work with other maternity care providers	Shared care is possible between OBs and FPs, depending on the care providers' comfort	RNs don't feel there are large impacts of their funding on MWs’ ability to work with other providers	OBs mostly work with FPs and MWs, not other OBs	Typical ways MWs work (e.g., consultations to physicians) are supported by the existing funding model, but there is desire for more collaborative models which doesn't work with current funding
	May be potential for lost income if care transferred from FP to OB but not a big concern	RNs don't always understand scope of practice of MWs; this can cause tensions after transfers of care (TOCs)	Easier to work with FPs than MWs because financial incentives are structured different	When a client moves or their care is transferred, there are differences in payment to MWs depending which type of providers a client sees
	In rural settings (particularly contexts where there are no Obs available), challenges arise when FPs doing maternity care bill OHIP for patients from a different family health team (FHT) as the other FHT gets negated for that visit, even if they do not provide that type of primary care	Differences in education and provider philosophy on provision of maternity care are seen as biggest challenge to interprofessional interaction	When OBs collaborate with MWs it can be challenging for collaboration with RNs; people unsure who should be doing what	Funding model is a barrier to collaboration with other health professionals
	Funding between OBs and FPs separate, so few impacts other than transfers of care, FP not always staying involved		Funding model is okay for being able to see patients and for consultations, but not collaboration	There is the potential for increased collaboration or role-sharing of MWs and physicians, but funding doesn't allow for it
			When OBs work with FPs both can bill for delivery; no penalization	Currently, lots of free labour happens; there is a need to not devalue work of MWs through this
			FPs quicker to transfer care to OBs than MWs because they can still bill for delivery; Obs perceive need for TOC to impact MWs more	Funding model is a barrier to creativity to address health system needs
Impacts of midwifery funding arrangements on your ability to work with them (or for midwives, impacts of physician funding arrangements)	Increasingly low-risk pregnant people are choosing midwifery care, so FPs seeing decreased volume of low-risk obstetrics to the point of potentially affecting feasibility and viability of providing care	When MWs are struggling with volume demands, hospitals are unable to implement novel approaches to address these challenges because of siloed funding arrangements	MWs as surgical assistants are beneficial and support continuity of care, but it may not be cost effective to have a midwife on standby for that task only	MWs pay is less than physicians; this hinders sense of equity between the professions
	Not possible to do shared care with midwifery because of differences in funding—not possible to divide up course of care without losing funding; "no one wants to work for free"	MWs' budgets and funding arrangements are not public information; lack of transparency is an issue	OBs still have to be available to the labour ward while MWs attend births; perception of babysitting	Some MWs have restrictions imposed by Obs on how much they can consult before a TOC is required; this impedes professional autonomy of MWs, interprofessional collegiality, and respect
	When there are examples of collaboration (for example in some FHTs), midwifery insurance costs present a barrier	Some RNs feel there are no impacts of funding arrangements	Some OBs perceive medicolegal concerns to be a bigger challenge than funding arrangements	Midwifery specific guidelines help legitimize the way MWs practice and supports them with evidence
	Ultimately collaboration very challenging because even with options for billing for consultations, FPs don't feel they are properly remunerated, or billing differences just present too much of a barrier		Frustration about multiple consults without a transfer of care because of the unremunerated work involved	Funding arrangements for MWs and physicians are a major barrier to multidisciplinary or inter-professional care
	Physicians and MWs work in parallel, not really possible to collaborate		Many MWs consult with OBs, not cost-effective	Funding differences means MWs are often not included in funding conversations in hospitals, which makes them be seen as second tier
	With FHTs, even if client is seeing a midwife who then consults with FP, FP doesn't get paid if client is originally theirs—only paid if they attend delivery		Feeling of being taken advantage by MWs because they are already at the hospital; MWs may not see patient before asking for a consult	
			No impacts with MFM	
			Lack of understanding of midwifery funding arrangements	
Unintended consequences of funding arrangements	Lower volumes of low-risk OB clients; declining volumes for family medicine means financial sustainability of providing this care challenging	System performance bench marking can affect funding allocation	Birth tourism places billing onus on provider; need for government regulation	Lack of flexibility of the midwifery model or ability to combine it with other models of care
	Funding arrangements haven't kept up with necessary changes to prenatal care that require more time	Health Based Allocation Model weighted cases are not always accurate, so intrapartum hospital care is not always appropriately funded; other factors should be considered in hospital funding allocations	Decreasing volume of deliveries per on-call shift over the last 15 years impacts income	For AMU, being funded separately means there is protection over MWs’ space and finances
	Funding of midwifery impedes ability to collaborate, innovative programs, integration of MWs into healthcare system	Increased funding for midwifery services may take away clients from OBs	Gender bias impacts funding of women's health	Presence of AMU means some MWs have never had experience working in other systems, find those systems very onerous
	Extra challenges with billing in rural areas as not all family doctors provide OB care, those that do end up taking funds away from the pregnant person’s primary FP	Reluctance to consult; discrepancies between providers about how many times to consult before TOC	Fee schedule doesn't necessarily reflect time or skill required for a procedure	Siloed funding means MWs are left out of many administrative conversations at hospitals
	C-sections and vaginal deliveries receive very similar payments, but sometimes C-sections are extra challenging or time-intensive, and physicians feel a bit frustrated that they are not adequately compensated for that	Nurse pushback to TOC because it increases their workload	Fee for service not helpful if not busy	In some settings, OBs/hospitals require MWs to transfer care under some circumstances where care remains within MWs’ scope; this leads to multiple billings for the same patient
		Lack of transparency about funding for MWs creates barriers and misperceptions	OBs don't personally know MWs at their hospital as well as they used to because of expanding practice and high turnover; no longer helping friends, helping strangers leads to tension	Need for MWs to consult with an FP or OB to arrange referral to some specialists increases cost to healthcare system and creates burden to pregnant person
		Billing arrangements present extra challenges for TOCs and when they should happen	OBs seen as a safety net for midwifery patients in hospital	Lack of strategic planning for the sector and not enough focus on money and who controls it, how, where; limited MWs integration into hospitals
		Clients and MWs experience disappointment when TOCs need to happen because it impedes involvement of midwifery	Some believe that expanding scope of practice of MWs erodes the work of OBs	Independent contractor model dictated much of current funding and working arrangements; this approach was originally preferred because of legal implications related to employment standards and on-call midwifery work
			Frustration because MWs get paid to care for uninsured patients, but OBs do not	Independent contractor model allowed for different places of birth
			Funding impacts satisfaction, but not delivery of services	There may be unintended consequences from shifting costs for surgical assistants between different funding sources (i.e., physician vs. midwife funding pools)
			Funded for sick care, not wellness; no funding for prevention and keeping people healthy because fee for service provides incentives to have visits with patients	
			Little incentive for OBs to support expanded scope of care for midwifery because "easier" things which MWs could do are easy for OBs to do and therefore easy money	
Solutions: Types of funding arrangements that would create the optimal delivery of maternity care in Ontario	Funding arrangement that would allow for shared care and shared income between FPs and MWs; collaborative funding model	Hospital funding based on percentage of maternity visits	Having MWs as surgical assistants would help with collaboration	Physician funding better suited to a salary model; some believe midwifery funding okay, but should be more options for consultations
			Hospitalist MWs would help reduce consults and assist new MWs	Salaried models facilitate interprofessional collaboration
		Creating quality-based procedures around maternity services	OBs need to be compensated for all consults, without limit and also compensated if present as a backup	More AMUs as they allow for autonomy but also integration within the system and allow for MWs to work in different, more flexible way
	Salary for MWs to work within family practices and provide maternity care	Funding for length of care provided and based on risk categories for people who are pregnant vs. billing based on volume of care and per conversation	Salary model; this would help avoid unnecessary TOCs and pay OBs even when they are not conducting births	Hybrid model with course of care as the core, but equitable FFS payments similar to physicians for other services above scope (but still within ability and competency)
	In rural settings, fee for service is okay, with additional maternity care things being "out of the basket"; need to avoid penalizing other providers for FPs who provide OB care caring for their clients however	MWs funded for all low-risk patients, can only see OB if high risk otherwise pregnant person has to pay	Truly coordinated care where multiple providers are available to pregnant people	Need for a hybrid model that is similar for both MWs and physicians; MWs should be allowed to bill some things FFS, or provide episodic care at times
	Blended models are ideal to avoid incentivizing volume or disincentivizing quality and care	Model where low-risk patients see midwifery, and higher risk-patients have a shared care model with Obs; model of collaboration vs. competition	A model which promotes interprofessional collaboration is ideal	Salaried models for OBs and physicians
		Financial incentives for hospitals to promote intra-professional collaboration	A model which remunerates antenatal care as much as deliveries	Need for a longer-term budget for maternity care; annualization doesn't work
			A combination model of fee-for-service and salaried positions	Need to ensure funding arrangements incentive working unusual ('unsociable') hours
			Pooled billings between OBs to even out differences in volume	Regionalization and funding relatively to hospital populations
			A capitation or roster model where you are paid for a certain number of patients—promotes health and prevention, instead of fee-for-service which pays for sickness or disease	Family doctors and MWs should provide all low-risk care, OBs just for high-risk or when a transfer is necessary because of risk
				Hospital On-Call Coverage funding could be really beneficial, especially in rural communities
				Need to restructure funding for MWs so it is not so complicated

### Impact of midwifery funding on midwifery integration

Participants identified several strengths of the CoC midwifery funding model, noting that it successfully supported its intended objectives of incentivizing continuity of care and spending time with clients, while also promoting the autonomy of midwives. Participants also noted that the funding model’s coverage of midwifery services for Indigenous communities on reserve and for residents of Ontario without health insurance were strengths, though it was also noted that provision of care for people without health insurance sometimes created interprofessional tension because of billing challenges for consulting physicians. The CoC funding model’s focus on continuity of care was seen as a positive factor that enhances patient experience and creates high satisfaction with midwifery care experiences:

*...all of the studies that I’ve seen, including the Maternity Experiences Survey that was done by the Public Health Agency of Canada, showed that there are higher rates of satisfaction with midwifery led care to physician led care. And again, I don’t think that’s about the person. I think it’s about the role and the kind of models that the funding is very directly related to kind of producing. So it doesn’t come as a surprise that someone in a midwifery model who is able to have half an hour or 45 minutes appointments and more appointments with a midwife may feel more satisfied than someone who has 5-minute appointments with a physician.* (PE1)

The continuity of care model that CoC funding supports was seen to have positive impacts at a system level in terms of cost containment through keeping patients with minor conditions out of the emergency department or obstetrical triage and enabling them to access timely information from their midwives.

*I see patients ... under midwifery care as well as family doctor and obstetrician, and we do see less hospital visits from the midwifery population and I know the argument can be made that they tend to be lower risk patients which makes sense, but they tend to have more access to their providers, their pregnancy and delivery providers for problems at home. They are able to call their providers. They are able to go into clinic for more minor things. Whereas patients under obstetricians and family doctors because of the way they are structured in their offices, they really don't have room for walk in and there is no access to those providers. So, those patients we then see then basically as an Emergency Room visit which is expensive.* (RN1)

Despite these strengths, participants noted several ways in which the CoC funding model limits midwifery integration. One key concern is that CoC based funding offers little flexibility as to the services for which midwives can be compensated (i.e., payment is only available for antepartum, intrapartum, and postpartum care, and only occurs if at least 12 weeks of care or attendance at birth is provided). Particularly in small communities, this is an impediment to creative solutions to ensure access to intrapartum care (e.g., midwives providing any elements of care to physicians’ patients). Midwives possess a range of skills that could be employed outside the context of a standard course of care (e.g., provision of postpartum care to physician patients) but there are limited mechanisms for them to be compensated to do so. Furthermore, in situations where the needs of a community change, midwives are not able to manoeuvre quickly and easily to answer those needs (e.g., to fill gaps created due to shortages of nurses or physicians). As one midwife noted, “*…there's been skills that we are capable of doing, that we are qualified to do, but that we haven't been able to do, because there's no mechanisms to get paid for it.”* (MW2).

Another key concern identified by several participants is that the incompatibility between FFS and CoC funding serves as a barrier to interprofessional collaboration. In discussing innovative ideas about how midwives, nurses and doctors might be able to work together to meet the needs of a community, the funding model posed an obstacle, and created little leeway on how caregivers might be paid to work together.^6^ As one family physician stated, “*…there is no way that given their funding on a case-to-case basis that we can truly share care with them or join ranks to provide, for example, intrapartum care*.” (FP1) In fact, several participants noted that instead of helping to build teamwork and cooperation, the current funding structures make midwives and physicians competitors. As this obstetrician remarked,

*The idea that you would have this funding model and somehow on some level think that we could work cooperatively over the long term as teammates, it doesn't work that way. The funding model simply creates too much tension for that to work. It just doesn't work. The funding model makes us competitors not team members.* (OB1)

The lack of alignment between maternity care funding models was identified as a challenge to economical care as a pregnant person might choose to move from their family doctor’s or obstetrician’s care to midwifery care late in pregnancy and therefore the cost of the antepartum care will have been essentially paid twice.^7^ This arrangement is not viewed favourably by some physicians and may create resentment towards midwives. As one participant described,

*...sometimes it’s even happened to some of us at 36 weeks that our patient has switched into midwifery care because of potentially them trying to take over our patients. Then that midwife is being paid for that entire expectation thinking that they’re taking care of the patient for the whole prenatal care experience, plus delivery, plus postpartum. So that’s like a real double-dipping.* (FP2)

Additionally, if a pregnant person requires obstetrical care in pregnancy and their midwife stays involved, two different practitioners are paid for the care. While similar arrangements exist to ensure that family physicians can bill for attendance at births when transfer of care to an obstetrician is required, the perception that work is being duplicated or that people are being paid for work they are not doing is a potential source of friction between midwives and physicians.

Finally, another key barrier to the integration of midwives is that midwifery funding is siloed separately from hospital and physician funding, which are themselves siloed from each other, and some of our physician and nurse interviewees had little understanding of the midwifery funding model. Siloed funding means that midwives are not considered as a feasible potential human resource to address gaps in services, nor are they considered in physician or hospital funding decisions that may affect them.

*We're left out of being sitting at those tables. We're not a consideration […] to fill a gap, because our funding is so vastly different […]. I think it creates a barrier when there's two completely separate systems. I think all of that affects patient health outcomes in – inadvertently, in a more subtle way than maybe a case-by-case way.* (MW2)

### Impact of physician funding on midwifery integration

One of the most important issues identified by participants related to the impact of physician funding on midwifery integration is that the growth of midwifery has a negative impact on remuneration for physicians. FFS physician funding arrangements incentivize volume, and as midwifery grows, physicians’ income from obstetrics will decrease as their volume of births decreases. This was identified across all participant groups as a significant barrier to midwifery integration.

*There truly is a barrier because physicians need a certain volume of births in order to maintain their status quo. [...] if midwives do more volume, even in middle or smaller volume settings, that it can undermine the stability of the rest of the maternity care environment. With fee-for-service systems […] they want a volume because of income.* (MW1)

In a more nuanced way, this tension reveals that current funding arrangements fail to appropriately remunerate obstetricians for consultative work. Both midwives and physicians identified inadequate OHIP remuneration for physicians being on call for intrapartum care, including availability for obstetrical emergencies and for intrapartum consultations from midwives. As one obstetrician noted, “*We are not making decisions, just sitting around waiting to be called. I'm pretty sure that fireman get paid even when they don't have to get their truck and go zooming somewhere. Right?” (OB1)* The current fee structure leads to frustration and tension as it undervalues the work of obstetricians.

*Let's just say I am the consultant, and the board is full of midwifery patients, and I am consulted on one of them to induce labour, and all of the other deliveries happen with their midwives. I've just spent 24 hours of my life, and I didn't get paid, even though I'm at the beck and call of midwifery to help them if there's a sudden urgent need for a C-section. My being there doesn't pay me. (OB4)*

An unintended consequence of these circumstances is that in some settings, obstetricians require unnecessary transfers of care from midwifery to obstetrics as a workaround to be appropriately paid for their work and perceived increased in liability.

*I think the one that comes to mind around physicians is it’s really concerning that there’s a very high rate of medically unnecessary transfers of care and there could be a number of reasons for that, but certainly as I understand them… that that’s one of the significant contributing factors in physician payment and so that it’s the physician payment piece. […what’s needed is a] funding model that would allow them to kind of be in that consultancy role so that transfers of care are not motivated by who’s getting paid or by the physician being paid, but rather by what the client or the patient needs.* (PE1)

In addition to increasing costs for the system that arise with two providers being paid for intrapartum care, unnecessary transfers of care from midwives to obstetricians represent an underutilization of the knowledge and skills of midwives.

Another barrier to the ideal integration of midwifery is that the OHIP Schedule of Benefits limits the type of physicians to whom midwives can make direct referrals. For example, endocrinologists and psychiatrists cannot bill OHIP for a consultation when a referral comes directly from a midwife. Instead, midwives must arrange for an additional patient visit with a family physician or obstetrician in order to access care from these specialists for midwifery clients. This creates inconvenience for midwifery clients and providers, may delay access to needed care, and creates unnecessary costs for the health system.

### Impact of hospital maternity care funding on midwifery integration

We found that hospital intrapartum care funding arrangements in Ontario, which are driven by both volume and acuity, may either encourage or discourage midwifery integration depending on contextual factors. In settings where there is opportunity to increase the volume of births at a hospital, the integration of midwives may be viewed as a benefit to a hospital because midwives provide intrapartum care that would otherwise be provided by nurses paid by the hospital and midwife-attended births have shorter lengths of stay on average and therefore lower direct costs to the hospital [40]. In other hospitals, intrapartum care is not viewed as a significant source of income for the hospital and therefore is not a priority, making changes to the status quo and the integration of midwives more difficult. In settings with higher birth volumes, the financial incentive to provide high risk care (i.e., high acuity) may lead hospitals to minimize the volume of births attended by midwives. As this participant described, there is not a consistent pattern in how hospitals have interpreted hospital funding to dictate the approach to integrating midwifery:

*I know some hospitals have really looked to midwifery as a way to increase their volume to support hospital funding in places where they need higher volumes as births. At the same time, I've seen hospitals really constrain the amount of midwifery births that happen at that hospital, because in the way they interpret the funding, or the way more high-risk centers are funded, there's a perception at least of disincentive to do low-risk births, that there's some priority given to extra financial incentive given to high-risk births. I think, because it's interpreted differently in different places.* (MW3)

One nurse participant noted that it is important to understand that shifting a large portion of low-risk births to midwifery will increase the acuity of patients that nurses care for, which affects the necessary nurse patient ratio.

Of note, the fact that the decision-making structure of hospitals is physician centred was identified by several participants as being an important factor that contributes to the challenges of midwifery integration. Given the control that the MAC has over decisions about hospital privileges, in some settings, physician interests as opposed to hospital funding arrangements may have a greater influence on hospital related factors impacting midwifery integration. It was observed that hospital policies regarding consultation and transfer of care from midwives to obstetricians or the number of midwives at a hospital may be primarily motivated by ensuring obstetrician control and income.

*It tends to be because the physician or the obstetrician maintains more power in that hospital …[midwives] don't always have the same voice at the table. They don't have the same integration into the hospital admin structure, so the physicians are able to often run the show. The chief of obstetrics has more say in the policies than the chief of midwifery, if there even is a chief of midwifery. Then, you end up with it looks like a policy related to safety, but when you really unearth it, I think its billing is the motivation more than anything, control, control and money.* (MW4)

The distinction between hospital funding and midwifery funding also contributes to nursing perceptions that impede midwifery integration. Nurses sometimes perceive the care of midwifery clients to be the sole responsibility of midwives rather than a responsibility of the entire hospital team, and this can lead to a sense of resentment among nursing staff when midwifery clients require obstetrical and nursing care.

*…it's really busy on an obstetrical unit, the obstetrician has done a consult, feels that there should be a transfer of care, then there is the push back of the nursing staff because now they have to assume the care of the patient. It's more workload. It's back to that ‘oh, you're dumping on me.’* (RN3)

### Other notable findings

We also identified several cross-cutting themes that impact midwifery integration, including entrenched gender inequity in the funding of women’s healthcare, the impact of underfunding on burnout, dependence on the goodwill of providers, and concerns about liability.

In general, women’s healthcare is less well remunerated than many other types of care within the Ontario SoB for physician compensation [41]. For example, the time requirements and complexity of early antepartum care, which includes offering genetic screening and obtaining consent for such testing is not reflected in the fees paid to physicians for this work. As one obstetrician noted, “*You can provide care for the entire pregnancy, and $80.00 of those $100.00 that you make for providing that care are on the day of the delivery.*” (OB4). Another noted that,

*...women’s health is underfunded across the board. So, for example, if I compare myself to an ophthalmologist, an ophthalmologist gets paid just maybe $30.00 less for doing a cataract surgery, which takes seven minutes than me doing a four-hour hysterectomy. So, there are great disparities in our funding reimbursement. *(OB3)

A third obstetrician pointed to long waiting lists for gynecological care and explained how many obstetrician-gynecologists have moved away from gynecology due to “*the monetary differences between those two halves of the specialty.”* (OB5).

Another example of gender inequity and the devaluing of women's healthcare discussed by participants was the recent Human Rights Tribunal of Ontario decision related to the discriminatory under-compensation of Ontario midwives by the MOH. As one participant explained,

*…so some of that gender bias is that often work associated with women, like some of the emotional or caring work that happens is kind of seen as something that is not highly valued or not as highly valued as other skills.* (PE1)

The impact of underfunding pregnancy-related work, which requires on call work and is inherently unpredictable in nature, contributes to burnout across provider groups and negatively impacts midwifery integration. As one midwife explained,

*I think having underfunded providers leads to all sorts of consequences, including provider burnout, lack of interprofessional respect, inability for providers - feeling like they have to do more work than maybe they can do in a healthy way... I think some midwives feel like they have to work more than they can do in a healthy way, and I think that could probably relate to physicians as well, that in a fee-for-service model it kind of incents volume over quality.* (MW3)

One obstetrician noted that preventing burnout is not only important to ensure good integration of midwives, but is essential to achieving patient satisfaction, good health outcomes and the containment of costs. Both midwives and obstetricians identified that because the system does not remunerate important work done, it counts on the goodwill of healthcare providers. As one obstetrician described,

*I think we're all in it for what's best for the patients, and if a midwife calls me to the room for there's a prolonged deceleration for example, and I go running - it's always three in the morning, so I go running out of my bed. I come to the room, and the heart rate has corrected itself. I have just flexed my muscles, ran to the room, and I got nothing for that. Am I going to bill a consult for stepping into the room for one minute to be there to help her just in case? I got nothing for that.* (OB4)

Finally, although participants were not asked specifically about funding of liability insurance, the question of liability was repeatedly alluded to, either implicitly or explicitly, by participants. The funding of liability insurance, with different levels of liability coverage and different insurers for different healthcare providers, was also seen as an obstacle to interprofessional co-operation. Although the hospital, nurses, physicians, and midwives are ostensibly all focussed on providing the best care possible, the differences in funding structures for liability insurance means that the evaluation of risk of medico-legal liability, even when only hypothetical, may at times pit midwives, nurses, and physicians against each other.

### Proposed solutions to improve midwifery integration

Participants articulated a variety of strategies to address the current barriers that funding arrangements create for the integration of midwifery care. Respondents identified a foundational need for a cohesive, flexible long-term vision for reproductive care in Ontario that recognizes some of the uncontrollable aspects of such care in its plan. As one participant stated,

*...so like a strategy for Ontario, which, for perinatal care, which we don’t have right now, and so that some of the issues that come up around funding are lacking, I think, like an overall, cohesive vision from the Ministry that’s evidence informed and that it’s kind of able to move forward, or kind of move Ontario in a different path in terms of the quadruple aim and seeing that improved.* (PE1)

There was also broad consensus on the need for new funding arrangements (e.g., alternative payment plans) that will ensure appropriate compensation for physicians who are on call for emergencies and as consultants for low-risk obstetrical providers. Many physician participants expressed openness to non-FFS funding models, including salary-based models. Participants shared a range of ideas related to potential models of care (including alongside midwifery units and collaborative interprofessional teams) that would optimize the use of midwives’ knowledge and skills. Some participants spoke to physicians and hospitals starting to see potential contributions that midwives might make to filling gaps or improving care (e.g., facilitating early discharge through the provision of home-based postpartum care for physician patients, or acting as the surgical assistant at caesarean births), and noted that current funding arrangements limit these contributions. A common underlying thread was that funding arrangements should be flexible enough that care models can be responsive to the needs of specific communities and the healthcare providers who care for them, and allow collaboration and teamwork when appropriate. As one obstetrician suggested,

*I would say that we’re all working in silos, and we shouldn’t all be working in silos... I think the funding model where we again, could be working together as healthcare teams providing the necessary expertise to the patients that need, it would achieve all of the aims that you’re looking at with the quadruple aim… a coordinated care approach, where you have midwives, nurses and obstetricians working together in a team and each doing what they are best-suited to do and advise on, makes a lot more sense to me than the siloed model we have right now.* (OB2)

Several participants noted that the existing midwifery model of care, which is based on continuity of care, supports excellent clinical outcomes and high levels of client satisfaction, and highlighted that friction occurs as a consequence of incompatible funding models. While the addition of more flexible funding to expand the contributions of midwives is needed, these participants did not advocate for completely abandoning the midwifery led continuity of care models. At the same time, funding to compensate midwives to work outside the CoC model was seen as having potential benefits both in enhancing the feasibility of midwives contributing their skills to address gaps in the system and in creating additional work options that would increase retention and work satisfaction for some midwives.

### Findings of documentary analysis

Table 2 shows the organizations whose websites we searched for the documentary analysis.

**Table 2:** Websites searched for documentary analysis

Name of organization	Web link
Association of Ontario Midwives	https://www.ontariomidwives.ca/
Ontario Medical Association	https://www.oma.org/
Canadian Medical Protective Association	https://www.cmpa-acpm.ca/en/home
Hospital Insurance Reciprocal of Canada	https://www.hiroc.com/
College of Midwives of Ontario	https://www.cmo.on.ca/
Colleges of Physicians and Surgeons of Ontario	https://www.cpso.on.ca/
Canadian Agency for Drugs and Technologies in Health	https://www.cadth.ca/
Canadian Institute for Health Information	https://www.cihi.ca/en
Provincial Council of Maternal and Child Health	https://www.pcmch.on.ca/
Ontario Ministry of Health	https://www.health.gov.on.ca/en/
Champlain Maternal Newborn Regional Program	http://www.cmnrp.ca/en/cmnrp/Home_p2974.html
Canadian Medical Association	https://www.cma.ca/
College of Family Physicians	https://www.ontariofamilyphysicians.ca/
Society of Obstetricians and Gynaecologists of Canada	https://sogc.org/
Maternal, Newborn, Child and Youth Network	https://mncyn.ca/
Ontario Hospital Association	https://www.oha.com/
Ontario Health	https://www.ontariohealth.ca/

Overall, our documentary analysis corroborated the findings from our interviews. The majority of websites we searched did not contain any documents related to midwifery funding and integration. The most relevant documents that we identified came from websites of the Association of Midwives [4, 18, 42, 43] and the College of Midwives of Ontario [44]. Below we share the key insights from our analysis of the websites and documents.

Importantly, our findings highlight what is not said. First, there is an absence of public documentation regarding the vision or plan for the sexual and reproductive care workforce. Despite the sizable portion of healthcare spending on reproductive care (birth accounts for 10% of hospitalization costs in Canada) [45], Ontario does not have a leading organization focussed on reproductive care. Instead, reproductive care is grouped with child and youth health, in the Provincial Council for Maternal and Child Health (PCMCH), and provincial activities in this area have avoided workforce planning [46]. The Ontario Ministry of Health has identified the need to ensure system sustainability by developing “a long-term capacity plan […] that identifies the right mix of services, health care workers, infrastructure and tools needed to ensure the equitable allocation of health care is attained in the province” [10]; however, the websites of the Ontario Ministry of Health (including the Health Workforce Branch), the new Ontario Health website and PCMCH do not contain any public documentation of a workforce or human resource plan for sexual and reproductive healthcare [47]. There is documentary evidence of some regional attempts at planning. For example, the Champlain Maternal Newborn Regional Program’s report on its capacity plan included human resource recommendations including an increase in midwifery care [48], yet does not indicate how to deal with integration issues. As well, the PCMCH has performed a gap analysis of maternal-newborn care in rural and remote areas [49], but has not produced a human resource plan for these regions.

Second, we found that despite midwives attending approximately 20% of the births in Ontario, midwives are still regularly excluded from content about reproductive care and primary care. Of the five maternal newborn regional networks designated by the PCMCH, only 2 have websites and both have little mention of midwifery and no apparent participation of midwives in their leadership structures [50, 51]. Projects on primary care reform and other MOH initiatives did not explicitly include midwives [10, 52]. Physicians are the dominant force as evidence by the fact that submissions regarding primary care reform were made to an MOH-Ontario Medical Association working group [42]. Several reports on safety in obstetrics explicitly exclude midwifery from the discussion [53, 54]. While the exclusion of midwifery from such reports can be attributed partially to the absence of midwifery data in the data sources being examined, it illustrates and reinforces the perception that midwives are not “part of the system”. Furthermore, the Better Outcomes Registry and Network (BORN) registry captures very robust midwifery data that could be accessed to inform reports and clinical and heath policy decisions [55]. The Ontario Hospital Association, in collaboration with the Association of Ontario Midwives and the College of Midwives of Ontario has developed a manual to facilitate integration of midwives into hospitals^8^[56]; however, the manual does not address the subtle and complex funding barriers identified by the participants in our study.

## Discussion

Our research is the first to focus solely on the role of funding arrangements on the integration of midwives into the health system and provides a more detailed exploration of how funding arrangements impact midwifery integration in Ontario than previous research. Our findings show that while midwifery CoC-based funding successfully promotes a midwife-led continuity of care model that supports very high levels of client satisfaction it poses a barrier to midwives in Ontario contributing to addressing community gaps in sexual and reproductive health services and sometimes is resented by physicians. Incompatibility between CoC and FFS funding impedes interprofessional collaboration. FFS physician funding for antepartum and intrapartum care incentivizes volume, contributes to competition for births between physicians and midwives, increases nursing workload when unnecessary transfers of care occur, and fails to appropriately compensate consultants for the on-call back-up that they provide to low-risk intrapartum care providers contributing to the difficulties of midwifery integration. Furthermore, the siloed nature of funding for midwives, physicians, and hospitals, particularly in the absence of a well-defined health workforce plan, leads to a context in which potential opportunities for midwives to address health system gaps are often not realized and midwives often have little voice in decisions which impact their level of integration.

Our findings align with previous research that has identified that funding issues are an important barrier to midwifery integration in Ontario [5–9], as well as research and policy statements pertaining to rural environments and other parts of Canada [46, 57–62]. Prior research has similarly identified that differences in funding arrangements prevent people from developing collaborative, interprofessional initiatives in antepartum, intrapartum, and postpartum care [6, 7, 9, 57], and contribute to the exclusion of midwives from primary care reform in Ontario [5]. In particular, FFS funding for physicians has been noted to discourage collaboration [7, 63], and foster competition [9]. The issue of unpaid work identified in our study has also been noted by others,[8] and contributes to tensions between midwives and physicians, as do concerns re: “double payment” [6]. Our findings concur with previous reports calling for flexible, harmonized funding mechanisms that support interprofessional collaboration and the ability to respond to unique community needs [7, 62]. The need for mechanisms to ensure appropriate compensation (decoupled from volumes) for consultants who support low-risk providers was also emphasized in a previous report [7]. Prior research has also noted the power differential between physicians and midwives within hospitals and how this negatively impacts elements of midwifery integration [6, 8, 9]. Our findings point to the fact that restrictions on hospital privileges that limit midwifery integration in many communities are frequently driven by the issue of loss of income of physicians who practice obstetrics when more midwives practice within a hospital. Similar tensions have been noted with the integration of nurse practitioners into the Ontario system [64, 65], particularly when the introduction of nurse practitioners encroached on “bread and butter” billings in emergency departments where physicians were not salaried [66]. Research on midwifery integration outside Canada, the integration of nurse practitioners, and collaborative care models has all identified FFS funding of physician care as a barrier to the integration of non-physician primary care providers [64, 66–71]. Finally, previous reports on maternity care in Canada consistently identified the need for funding that supports a system that can respond flexibly to community needs [46].

Our findings also offer some new insights into how funding arrangements impact the integration of midwifery. First, several participants spoke of how current funding arrangements limit the additional contributions that midwives might make beyond the care reimbursed by the CoC funding model. This idea has not been a key finding of previous research, and we hypothesize that it may have been influenced by recent innovation in midwifery funding. In 2018, two years prior to when we conducted our interviews, Ontario introduced an alternative funding mechanism that has supported a very small percentage of midwives to work in ‘expanded midwifery care models’ which are primarily salary-based [72]. This funding has allowed new roles to be created for midwives, including within hospitals and in primary healthcare teams, and has demonstrated that there are opportunities for midwives to contribute their knowledge and skills to address gaps in available sexual and reproductive healthcare outside of, or in addition to, the typical Canadian model of midwifery care. As an extension to this theme, some participants also spoke to hospitals starting to see potential contributions that midwives might make to filling gaps or improving care. However, while there is nothing to prevent hospitals from using their funding to hire midwives to provide care, doing so would entail shifting costs previously born by midwifery or physician funding pools to hospital budgets, so historic funding arrangements are a barrier to midwives being integrated into hospital care in new roles. Second, many of our physician participants expressed openness to non-FFS funding models for antepartum and intrapartum care, particularly as a solution to address the need for appropriate compensation for specialist consultants; this may reflect a new level of readiness for funding innovation. Our research was not designed to assess provider group funding model preferences but suggests that these may be worth exploring. Recent Canadian research beyond the field of pregnancy care suggests that a notable proportion of specialists are interested in alternatives to FFS and suggests there may be untapped opportunity to shift to new funding models for physicians [73].

### Strengths and limitations

Our research has several strengths. First, our interprofessional research team helped to facilitate broad recruitment of participants and to minimize bias in how we interpreted our findings. Second, examining a single context allowed us to conduct an in-depth exploration of how funding arrangements impact midwifery integration. Finally, our use of both interview and documentary data supported data triangulation.

There were also some limitations. First, we did not include healthcare service users as participants. Given our objective of understanding how and why funding arrangements impact midwifery integration, we chose to interview participant groups who would have the greatest familiarity with the details of existing funding arrangements. Nonetheless, healthcare service users may have offered a distinct perspective on the impact of funding on the integration of midwifery that we have not captured. Second, while we intentionally invited participants across a range of different settings and continued interviews until we reached data saturation, it is possible that we have missed some opposing viewpoints or alternative explanations. We did not collect detailed demographic data to examine this systematically. We relied on the diverse experiences of our interprofessional research team to minimize this limitation as best as possible. Third, our documentary analysis was limited to publicly available documents. It is possible that other relevant private documents exist that we were unable to identify.

### Implications for policy

Our findings suggest the need for three key policy changes to support improved midwifery integration and make optimal use of the scopes of all healthcare providers. First, new funding arrangements that ensure adequate remuneration for obstetricians for being on call and available for consultation, possibly through alternative payment plans, are needed to appropriately compensate them for their important consultative role as experts in obstetrical complications and emergencies. Moving away from physician remuneration that solely rewards volume of service and thereby incentives unnecessary transfers will be necessary to alleviate barriers to midwifery integration that are created when there is interprofessional competition for volume. Second, an expansion of more flexible funding arrangements that support midwives to utilize their full skillset and meet community needs for care that does not fall within the traditional course of care or for care that is episodic is required. Ideally this should include funding that can be used by hospitals to hire midwives. The expansion of flexible midwifery funding arrangements should incorporate funding arrangements that incentivize and protect continuity of care given the evidenced-based benefits of such models. Third, there is a need for a comprehensive and clearly articulated vision for antepartum, intrapartum, and postpartum care, including a human health resources plan, in the province that can be used to guide the development of equitable and flexible funding policies which will support the quadruple aim.

## Conclusion

Despite agreement between regulatory bodies and professional associations regarding the need for integration, trust, and respectful interprofessional collaboration and collegial relationships [43, 44, 62], midwives are still not ideally integrated into the healthcare system in Ontario. Siloed funding for midwives, physicians, and hospitals hampers the integration of midwifery. Policy changes that ensure adequate remuneration for intrapartum consultants, create flexibility to compensate midwives for care beyond the traditional course of care, and lay out a clearly articulated health human resource plan for sexual and reproductive healthcare are needed to improve midwifery integration and achieve the quadruple aim.

### Supplementary Information

**Additional file 1. **Interview guide 

## Data Availability

All data contributing to the analyses in this study are stored on a secure network and not publicly available in order to protect participant confidentiality. Enquiries regarding the data should be directed to the corresponding author, Dr. E.K. Darling.

## Abbreviations

AMU: Alongside Midwifery Unit
BORN: Better Outcomes Registry and Network
CoC: Course of care
FFS: Fee for service
LHIN: Local health integration network
MOH: Ontario Ministry of Health
OH: Ontario Health
OHIP: Ontario Health Insurance Plan
OMCEP: Ontario Maternity Care Expert Panel
OMP: Ontario Midwifery Programme
PCMCH: Provincial Council on Maternal and Child Health
SoB: Schedule of Benefits

## Footnotes

1 Hospital privileges are appointments to a hospital which also specify the procedures that an individual may perform in the hospital. In Ontario, physicians, dentists, and midwives may obtain hospital privileges.

2 Many family physicians are paid through a capitation model, but obstetrical care is remunerated separately by FFS. Obstetricians in academic centres may also be paid partially through an alternative payment plan to recognize the additional academic tasks that are taken on.

3 Of note, the majority of family physicians who do obstetrics do not receive any stipend for on call work for obstetrics. Most obstetricians do.

4 The midwifery model of care in Ontario includes: the client as primary decision-maker, informed choice, choice of birth place and continuity of care. As well the midwifery model of care may include early discharge from hospital and care in the community with use of hospital services on an outpatient basis.

5 This interview guide was pilot tested on a panel of researchers and midwives.

6 Since the time of these interviews there has been an alternative funding mechanism in place which currently funds very few projects. Expansion of this funding mechanism may resolve the issue discussed.

7 Of note, there are also situations in which midwives provide < 12 weeks of care to an individual and are therefore not paid for any of the care provided.

8 At the time of publication, this manual was under review.
