# Supplementary material for: The impact of funding models on the integration of Ontario midwives: a qualitative study
Source: BMC Health Serv Res. 2023 Oct 11;23:1087. doi: 10.1186/s12913-023-10104-7 (PMC10568882; doi:10.1186/s12913-023-10104-7)
Supplement: Supplementary file 1 — Additional file 1. Interview guide [file 12913_2023_10104_MOESM1_ESM.pdf]

Preliminary interview guide

Title of study:	Understanding the impact of funding arrangements on midwifery integration in Ontario

Ethical considerations
A description of the study will have been presented during the recruitment phase. Signed consent will be obtained prior to engaging in the questions. Any ethical issues arising will be addressed prior to the first question and will be documented by the interviewer.

Process
Interviews will be recorded on a digital audio device, transcribed, and uploaded into a qualitative software program. Handwritten notes will also be made by the interviewer into her field notebook.

✓ Denotes probes

Date: ______________________________

Time: ______________________________

Place: ______________________________

Interviewer: ______________________________

Interviewee: ______________________________

Position/role of interviewee: ______________________________

Questions for family physicians

1) Do you have any questions for me before proceeding to the interview?

2) Please provide a high-level description for me of how you are funded to do the clinical work you do.

3) How do your funding arrangements impact how you work with other providers of primary maternity care (e.g., nurses, midwives, and obstetricians)?
    a. How do the funding arrangements of midwives impact your ability to work with them?

Often in healthcare we're interested in the health system 'quadruple aim', which refers to improving population health and, patient and provider experiences, while keeping per capita costs manageable.

4) How do you think funding arrangements for maternity care impact the quadruple aim?
    - Access to care?
    - Patient health outcomes?
    - Patient experience (e.g., satisfaction, communication and coordination of care)?
    - Satisfaction as a provider of primary maternity care?

5) What are some of the unintended consequences of your funding arrangements?
    a. How do your funding arrangements create incentives or disincentives to focus more on providing certain services within your scope than others?
    b. Are there ways in which your funding arrangements impacts clinical decisions?
    c. Are there ways in which funding arrangements influences which providers of primary maternity care and how many are included in your hospital?

6) In an ideal world, what funding arrangements would be optimal for the delivery of maternity care in Ontario?
    a. What funding arrangements would facilitate collaboration?
    b. What funding arrangements would facilitate best access to care for patients?

Questions for midwives

1) Do you have any questions for me before proceeding to the interview?

2) Please provide a high-level description for me of how you are funded to do the clinical work you do.

3) How do your funding arrangements impact how you work with other providers of primary maternity care (e.g., family physicians, nurses, and obstetricians)?
    a. How do physician funding arrangements impact your ability to work with them?

Often in healthcare we're interested in the health system 'quadruple aim', which refers to improving population health and, patient and provider experiences, while keeping per capita costs manageable.

4) How do you think funding arrangements for maternity care impact the quadruple aim?
    - Access to care?
    - Patient health outcomes?
    - Patient experience (e.g., satisfaction, communication and coordination of care)?
    - Satisfaction as a provider of primary maternity care?

5) What are some of the unintended consequences of your funding arrangements?
    a. How do your funding arrangements create incentives or disincentives to focus more on providing certain services within your scope than others?
    b. Are there ways in which your funding arrangements impacts clinical decisions?
    c. Are there ways in which funding arrangements influences which providers of primary maternity care and how many are included in your hospital?

6) In an ideal world, what funding arrangements would be optimal for the delivery of maternity care in Ontario?
    a. What funding arrangements would facilitate collaboration?
    b. What funding arrangements would facilitate best access to care for patients?

Questions for nurses

1) Do you have any questions for me before proceeding to the interview?

2) Please provide a high-level description for me of how you are funded to do the clinical work you do.

3) How do your funding arrangements impact how you work with other providers of primary maternity care (e.g., family physicians, midwives, and obstetricians)?
   a. How do the funding arrangements of midwives impact your ability to work with them?

Often in healthcare we're interested in the health system 'quadruple aim', which refers to improving population health and, patient and provider experiences, while keeping per capita costs manageable.

4) How do you think funding arrangements for maternity care impact the quadruple aim?
   - Access to care?
   - Patient health outcomes?
   - Patient experience (e.g., satisfaction, communication and coordination of care)?
   - Satisfaction as a provider of primary maternity care?

5) What are some of the unintended consequences of your funding arrangements?
   a. How do your funding arrangements create incentives or disincentives to focus more on providing certain services within your scope than others?
   b. Are there ways in which your funding arrangements impacts clinical decisions?
   c. Are there ways in which funding arrangements influences which providers of primary maternity care and how many are included in your hospital?

6) In an ideal world, what funding arrangements would be optimal for the delivery of maternity care in Ontario?
   a. What funding arrangements would facilitate collaboration?
   b. What funding arrangements would facilitate best access to care for patients?

Questions for obstetricians

7) Do you have any questions for me before proceeding to the interview?

8) Please provide a high-level description for me of how you are funded to do the clinical work you do.

9) How do your funding arrangements impact how you work with other providers of primary maternity care (e.g., family physicians, midwives, and nurses)?
   a. How do the funding arrangements of midwives impact your ability to work with them?

Often in healthcare we're interested in the health system 'quadruple aim', which refers to improving population health and, patient and provider experiences, while keeping per capita costs manageable.

10) How do you think funding arrangements for maternity care impact the quadruple aim?
   - Access to care?
   - Patient health outcomes?
   - Patient experience (e.g., satisfaction, communication and coordination of care)?
   - Satisfaction as a provider of primary maternity care?

11) What are some of the unintended consequences of your funding arrangements?
   a. How do your funding arrangements create incentives or disincentives to focus more on providing certain services within your scope than others (e.g., gynecology care vs. obstetrics)?
   b. Are there ways in which your funding arrangements impacts clinical decisions (e.g., non-indicated transfers of care)?
   c. Are there ways in which funding arrangements influences which providers of primary maternity care and how many are included in your hospital?

12) In an ideal world, what funding arrangements would be optimal for the delivery of maternity care in Ontario?
   a. What funding arrangements would facilitate collaboration?
   b. What funding arrangements would facilitate best access to care for patients?
